# Peroxiredoxin II Regulates Cancer Stem Cells and Stemness-Associated Properties of Cancers

**DOI:** 10.3390/cancers10090305

**Published:** 2018-09-03

**Authors:** Nisansala Chandimali, Dong Kee Jeong, Taeho Kwon

**Affiliations:** 1 Laboratory of Animal Genetic Engineering and Stem Cell Biology, Advanced Convergence Technology and Science, Jeju National University, Jeju 63243, Korea; nimminisha28@gmail.com; 2 Laboratory of Animal Genetic Engineering and Stem Cell Biology, Subtropical/Tropical Organism Gene Bank, Jeju National University, Jeju 63243, Korea

**Keywords:** cancer stem cells, stemness, reactive oxygen species, Peroxiredoxin II, oxidative stress

## Abstract

Cancer stem cells (CSCs) represent a sub-population of cancer cells with the ability to regulate stemness-associated properties which are specifically responsible for unlimited growth of cancers, generation of diverse cancer cells in differentiated state and resistance to existing chemotherapy and radiotherapy. Even though, current therapies destroy majority of cancer cells, it is believed to leave CSCs without eradicating which may be the conceptualization for chemoresistance and radio-resistance. Reactive oxygen species (ROS) maintain stem cells and regulate the stemness-associated properties of cancers. Beyond the maximum limit, ROS can damage cellular functions of cancers by subjecting them to oxidative stress. Thus, maintenance of ROS level plays an important role in cancers to regulate stemness-associated properties. Peroxiredoxin II (Prx II) is a member of peroxiredoxin antioxidant enzyme family which considers as a regulator of ROS in cellular environments by modulating redox status to maintain CSC phenotype and stemness properties. Prx II has cell type-dependent expression in various types of cancer cells and overexpression or silenced expression of Prx II in cancers is associated with stem cell phenotype and stemness-associated properties via activation or deactivation of various signaling pathways. In this review, we summarized available studies on Prx II expression in cancers and the mechanisms by which Prx II takes parts to regulate CSCs and stemness-associated properties. We further discussed the potential therapeutic effects of altering Prx II expression in cancers for better anticancer strategies by sensitizing cancer cells and stem cells to oxidative stress and inhibiting stemness-associated properties.

## 1. Introduction

Peroxiredoxins (Prxs) are an important superfamily of small non-seleno peroxidases [1]. Members of the Prx family are divided into three classes, depending on the numbers of conserved cysteine (Cys) residues participating in redox reactions. Those classes, typical 2-Cys Prxs (Prx I, II, III and IV), atypical 2-Cys Prx (Prx V) and 1-Cys Prx (Prx VI) [2] are essential for intracellular reactive oxygen species (ROS) maintenance by scavenging hydrogen peroxide (H_2_O_2_) and organic hydroperoxide [3]. Imbalance of ROS and oxidative stress have been reported to contribute to cancer initiation by increasing DNA mutations and to cancer progression by activating signaling pathways involved in malignant transformation [4,5]. Among those, H_2_O_2_ has been known as a key signaling molecule in redox signaling that regulates multifarious signaling pathways involved in cellular processes such as proliferation, autophagy, differentiation, migration, metastasis, angiogenesis, DNA damage, inflammation and drug resistance of cancers [6,7,8]. Thus, Prxs are believed to play important roles as regulators of redox signaling in carcinogenesis [9] and thereby therapeutic targets for several including lung [10,11], colon [12], prostate [13,14], ovarian [15] and glioblastoma cancers [16], because overexpression of Prxs is considered to protect those cancer cells.

Among Prx family members, 2-Cys Prx enzymes catalyze H_2_O_2_ reduction to water more efficiently than other Prx members, by utilizing NADPH-donated electrons via glutathione-glutathione reductase system [17]. One of the typical 2-cysteine Prxs, Prx II is frequently upregulated in many cancers including breast [18], colon [19], prostate [20], lung [21], and liver [22] and downregulated in cancers such as melanoma [23] and gastric cancers [24]. Those Prx II expressions have been reported to involve in tumor progression, lymph node metastasis, signaling, sensitivity of cancer cells to radiation and therapeutic drugs in various types of cancers [25,26,27]. Moreover, Prx II maintains the survival of tumors by protecting cells against ROS injury and apoptosis as an important member of ROS scavenging system [12].

Cancer stem cells (CSCs) have been identified in number of cancer types [28]. Mainly CSCs are responsible for stemness-associated properties including self-renewal, differentiation, tumor progression, epithelial-mesenchymal transition (EMT), metastasis, expression of stemness genes and resistance for existing chemotherapy and radiotherapies [29,30]. Prx II is capable to maintain CSC phenotype and to induce stemness-associated properties [22,31]. Production of ROS is higher in tumor microenvironment, therefore, CSCs exclusively sustain antioxidant mechanisms such as peroxiredoxin enzymes system to detoxify elevated levels of ROS and to maintain redox balance [32]. It results downregulated ROS in CSCs and causes CSCs more resistant to oxidative stress and conventional cancer therapies [32,33,34] Therefore, eradication of CSC population remains a challenge to overcome for a successful clinical management of cancer patients. Therapies, which target Prx II may enable specific CSC targeting strategy to eradicate cancers.

**Figure 1:**
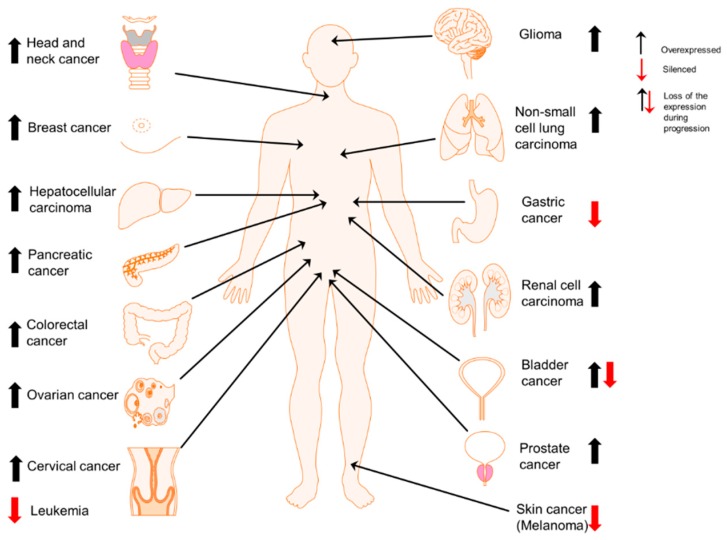
Cell type-dependent expression of Prx II in cancers. Overexpression of Prx II in majority of cancer types including head and neck [25], breast [35], liver (hepatocellular carcinoma) [22], pancreatic [36], colorectal [12], ovarian [37], cervical [38], brain (glioma) [39], lung (non-small cell lung cancer) [21], kidney (renal cell carcinoma) [40] and prostate cancers [20] and epigenetically silenced expression of Prx II in gastric cancer [24], melanoma [41] and leukemia [5]. Specially, in bladder cancers, overexpressed Prx II is significantly reduced with the cancer progression [42].

In this review paper, we summarized the current understandings on Prx II expression in multifarious cancers (Figure 1) and mainly the mechanisms by which Prx II maintains CSC phenotype and involves in stemness-associated characteristics and redox balance of those cancers. We also highlighted the possibility of Prx II downregulation or overexpression to use as a potential therapeutic target for cancers by inhibiting stemness-associated properties.

## 2. Prx II Maintains Cancer Stem Cell Properties of Hepatocellular Carcinoma via VEGF/VEGFR/STAT3 Signaling and Ras/FoxM1 Signaling

**Figure 2:**
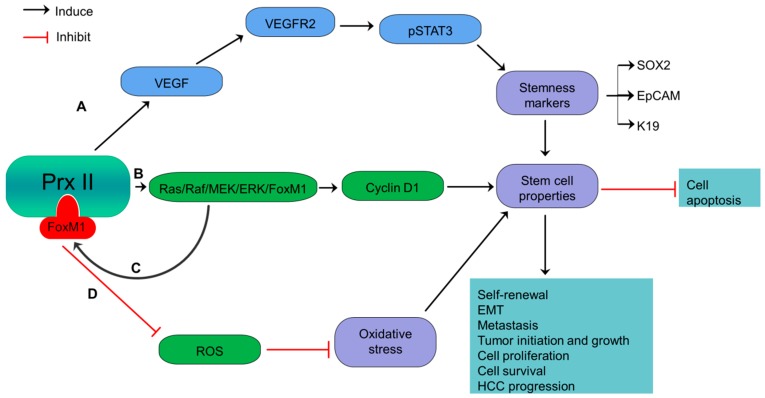
Overexpressed Prx II regulates stemness-associated properties of HCC cells. (**A**) Activation of VEGF/VEGFR2/STAT3 signaling via overexpressed Prx II-mediated stimulation of VEGF, to induce stemness gene expression such as SOX2, EpCAM and Keratin 19 and maintenance of stem cell properties. (**B**) Inducing Cyclin D1 by overactivation of Ras/Raf/MEK/ERK/FoxM1 pathway to induce stemness properties of HCC. (**C**) Additionally, the transcriptional activation of Prx II by overexpressed FoxM1 transcription factor. (**D**) Inhibition of oxidative stress and upregulation of stemness-associated properties by targeting ROS.

Hepatocellular carcinoma (HCC) is the most common primary liver cancer which causes majority of cancer-related deaths worldwide [22]. Prx II is significantly up-expressed in HCCs and hepatitis B virus related liver fibrosis which can develop to HCC. Prx II is reported to prevent TNF-α-induced apoptosis and programmed necrosis of HCC cells [22,43,44,45]. Moreover, high Prx II expression induces the stemness of HCC stem cells by inhibiting ROS-induced cell death [22,44]. High expression of Prx II in HCC also induces the self-renewal capacity, EMT, metastasis, cell survival, angiogenesis and stemness of CSCs via activation of VEGFR2/STAT3 signaling by vascular endothelial growth factor (VEGF) stimulation (Figure 2) [22]. Prx II protects redox-sensitive VEGFR2 tyrosine kinase activities against H_2_O_2_-mediated inactivation. A study reported that the absence of Prx II causes VEGFR2 inactivation by elevated H_2_O_2_ level. Therefore, Prx II is considered as most specific antioxidant regulator of VEGF-VEGFR2 signaling by promoting VEGF-induced tyrosine phosphorylation [46]. Partially overlapped localization of Prx II and VEGFR2 in caveolae plays an important role in Prx II-mediated protection of VEGFR2 against oxidation by preserving the reduced state of oxidation-sensitive cysteine residues of VEGFR2 tyrosine kinase [46].

Moreover, another study has reported that Prx II promotes H-ras driven hepatic carcinogenesis via activating Ras/Raf/MEK/ERK/FoxM1/cyclin D1 signaling pathway [47]. Many cellular functions in cancers, including cell survival, differentiation and proliferation are regulated by oncogenes of Ras family including H-ras [22]. Development and progression of HCC also are reported to associate with activation of Ras/Raf/MEK/ERK pathway and elevated levels of Cyclin D1 as a downstream signaling of ERK signaling [47,48]. Prx II positively regulates the activities of H-ras and subsequently activates Raf/MEK/ERK cascade [47]. FoxM1, a transcription factor with similar expression pattern to Prx II and capability to protect cancer cells from oxidative stress has been identified to govern Prx II overexpression via transcriptionally activating Prx II in HCC cells. On the other hand, Overexpressed Prx II positively associates with FoxM1 up expression via ERK signaling [47]. Therefore, hepatic carcinogenesis is driven by Prx II via H-ras/Raf/MEK/ERK/FoxM1/Cyclin D1 axis [47].

Moreover, upregulated Prx II correlates with the expression of HCC-related stemness markers such as epithelial cell adhesion molecule (EpCAM) and keratin 19 and induces tumor initiation abilities [49]. Thus, Prx II is considered as a candidate biomarker to target HCC stem cells in the therapies to prevent hepatocarcinogenesis [47]. A previous study has introduced adenanthin as a natural compound to kill HCC cells by directly targeting Prx II [50].

## 3. Inverse Relationship of Prx II Expression and Stemness Characteristics of Gastric Cancers

Gastric cancer is the fourth most commonly diagnosed cancer and is considered to be the third most common-cause of cancer related deaths in the world [51]. Recently, a study on gastric cancer cells showed the low levels of protein and mRNA expression of Prx II in gastric cancers. Furthermore, the same study revealed that the expression of Prx II is epigenetically silenced in gastric cancer cells [24]. CPG methylation of Prx II gene promoter causes silencing of Prx II gene in gastric cancer cells, because the methylations of CPG islands lead to transcriptional silencing of their downstream genes [52]. Prx II is one of major scavengers of ROS in cancer cells. Therefore, silencing of Prx II gene results elevated ROS level, as the downregulation of Prx II is likely to be the main cause for ROS elevation in gastric cancers [24].

**Figure 3:**
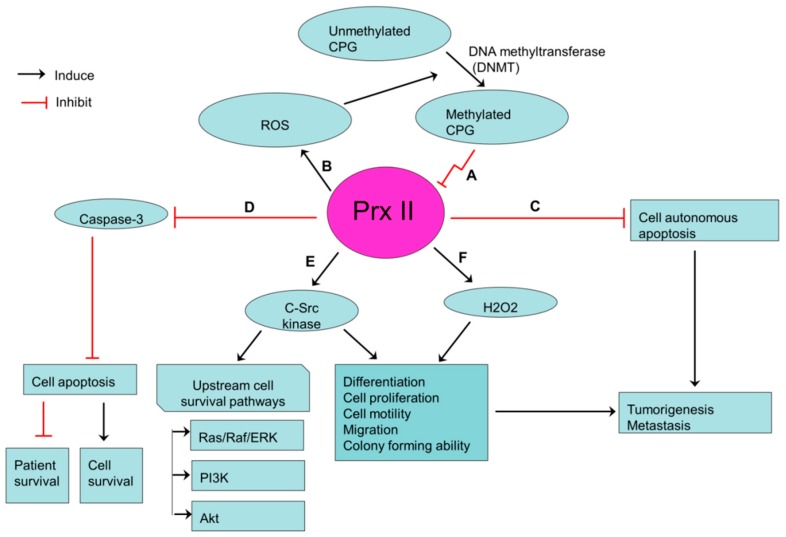
Prx II-mediated stemness-associated properties in gastric cancers. (**A**) Transcriptionally silenced expression of Prx II in gastric cancer cells by hypermethylation of CPG islands in promoter regions of Prx II. (**B**) ROS level is increased by silenced Prx II and Increased ROS induces the hypermethylation of CPG islands. (**C**,**D**) Reduced cell autonomous apoptosis and apoptosis via inhibiting caspase activities by silenced Prx II. (**E**,**F**) Over activation of c-Src kinase and H_2_O_2_ level by silenced Prx II, which then promotes the stemness-associated properties.

According to the positive feed-forward regulatory loop proposed by Hong et al. [24], increased ROS level causes upregulation of DNA methyltransferase (DNMT) expression and activities, which thereby elicits active transcriptional silencing of Prx II in gastric cancer cells by CPG methylation [53]. Low Prx II level in gastric cancers frequently associates with cancer stem like properties including cell proliferation, differentiation, tumorigenesis growth, colony formation, and metastasis [24]. Also, epigenetically silenced Prx II promotes tumorigenesis by inducing cell proliferation and differentiation in gastric cancers via elevated basal activation of c-Src kinase. C-Src kinase is highly activated in gastric cancers [24,54]. Induced Src activation affects gastric cancer cell motility and migration by modulating cell-cell or cell-matrix contacts such as adherent junctions, tight junctions and focal adhesion. Moreover, Src activation by silenced Prx II is a critical step for the activation of upstream cell survival pathways such as Ras/Raf/ERK pathway, PI3K and Akt pathways [55]. Induction of H_2_O_2_ expression through the silenced Prx II also increases the migrative ability of gastric cancer cells [56,57]. Survival of gastric cancer cells is increased by reduced caspase-3 activation and apoptosis activities, while reducing the survival of cancer patients by silenced Prx II (Figure 3) [24]. Furthermore, YO et al. [58]. showed the capability of Prx II antisense to induce cisplatin-induced gastric cancer cell-death and suggested that Prx II antisense may enhance the other anticancer-induced cell death, especially the drugs which are targeting ROS in gastric cancers [58].

## 4. JNK Activation is Induced by Aberrant Prx II Expression in Lung Cancers

Lung cancer is the most common type of malignancy and leading cause of cancer-related mortality in the world [59]. Small cell lung carcinoma (SCLC) and non-small cell lung carcinoma (NSCLC) are the two main histological types of lung cancers [60]. Prx II is reported to express highly in SCLC cells compared with normal lung epithelial cells and its elevated expression is associated with SCLC progression by inducing cell proliferation and inhibiting cell apoptosis [61]. Also, few years ago we showed that, Prx II is highly expressed in 3 different NSCLC cell lines. But the expression of Prx II is silenced in one of NSCLC cell lines (A549) due to the methylation in 5′-CCGG-3′ site. Interestingly, gefitinib-resistant derivative of A549 (A549/GR) only showed higher expression of Prx II [21]. Gefitinib is one of the inhibitors of epidermal growth factor receptor (EGFR) tyrosine kinase which is used as front-line treatment for NSCLC patients [41]. Demethylation of 5′-CCGG-3′ methylation site by gefitinib was identified as the reason for Prx II expression in A549/GR cells [21].

**Figure 4:**
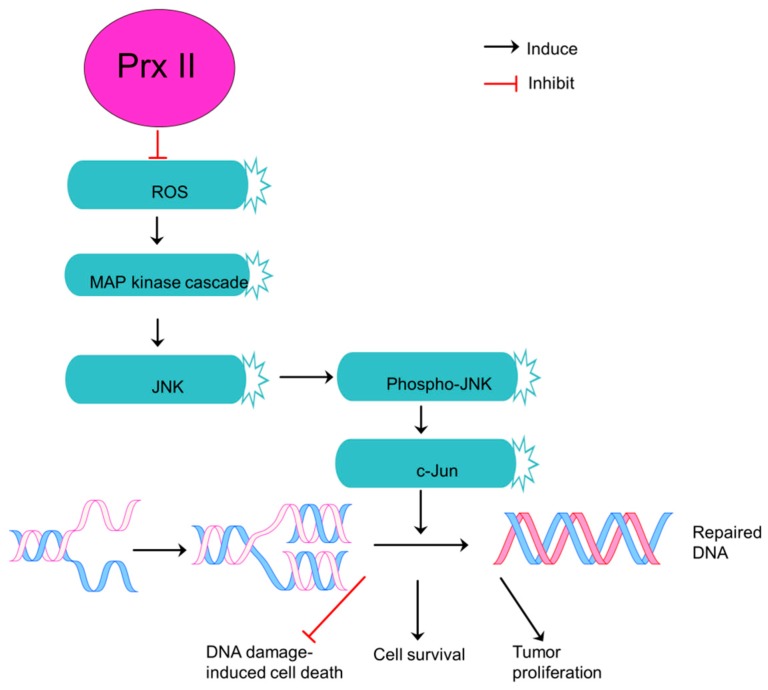
Prx II is an essential component of JNK-dependent survival pathway in non-small cell lung cancers. Downregulation of ROS level in NSCLCs by overexpressed Prx II and thereby activation of MAP kinase cascade to induce phosphorylation of JNK and JNK/c-Jun survival pathway which is essential to repair DNA damages. Repaired DNA-damages protects NSCLC cells from DNA damage-induced cell death and induces cell survival and tumor proliferation.

Highly expressed Prx II in A549/GR cells induces cell proliferation and tumor progression via activation of c-Jun N-terminal kinase (JNK) signaling and inhibition of apoptosis signaling (Figure 4) [21]. JNK signaling pathway is activated by mitogen-activated protein kinases (MAPKs) [62] which consider as proteins responsible for regulation of cellular events such as cell survival, cell proliferation and differentiation in NSCLCs. JNK pathway also is indispensable for the completion of DNA repair in cancer cells including NSCLCs [21]. Interestingly, Prx II positively regulates JNK activation and JNK-dependent DNA repairing process in nuclei thereby selectively protecting cancer cells against DNA damage-induced deaths. Thus, Prx II allows A549/GR cells to survive under DNA damages [63]. Prx II minimizes the sensitivity of cancer cells including NSCLC cells, A549/GR cells to DNA-damaging therapeutic agents through regulation of DNA repair [21,63]. But, the forced downregulation of Prx II expression inhibits JNK-dependent DNA repair and sensitizes cancer cells to therapeutic agents. Therefore, Prx II is considered as a novel therapeutic target for lung cancers.

## 5. Prx II Expression Level is Inversely Related to Stemness Characteristics and Cellular Activities of Malignant Melanoma

Melanocytes-derived malignant melanoma is considered to be the most aggressive type of skin cancers, because of its poor prognosis even after surgery [64]. Several studies have reported that Prx II expression in melanoma is relatively low compared with the other cancer types and normal melanocytes. According to those studies, hypermethylated putative promoter CPG islands (CGIs) of Prx II causes for silenced level of Prx II expression in malignant melanoma [23,65,66,67,68]. Prx II is considered to be a key antioxidant enzyme which regulates intracellular H_2_O_2_ level in melanoma cells [67]. But, silenced Prx II expression level fails to maintain that constitutive level of H_2_O_2._ Moreover, it modulates antioxidant activity in metastatic melanoma, thereby stimulating melanoma cell growth and increasing oncogenic potential through activating kinase signaling [69,70].

**Figure 5:**
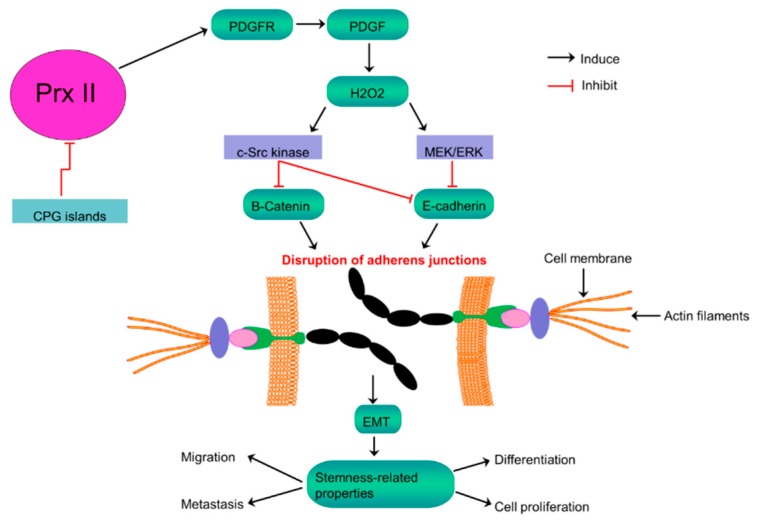
Inversely related Prx II expression and stemness-associated properties in melanoma cells. Activation of PDGF signaling by epigenetically silenced Prx II in melanoma cells to induce H_2_O_2_ level results consequent over activation of c-Src kinase and MEK/ERK signaling to inhibit E-Cadherin and the membrane retention of β-Catenin, thereby disrupting adherens junctions to promote EMT and stemness-associated properties of melanoma cells.

Intracellular H_2_O_2_ is also produced in cells as response to a variety of extracellular stimuli including platelet-derived growth factor (PDGF), a peptide growth factor [71]. As the main and specific negative regulator of PDGF signaling, Prx II suppresses phosphorylation of PDGF, by interacting with activated PDGF receptor (PDGFR) [65]. But, this suppression of PDGF phosphorylation is abolished by the silenced level of Prx II in melanoma by augmenting PDGF signaling and following induced production of H_2_O_2_. Elevated expression of H_2_O_2_ enhances the activation of PDGFR and phospholipase C_γ_1 [65,67,72]. Accumulated H_2_O_2_ promotes the activation of proto-oncogene tyrosine-protein kinase Src (c-Src) and extracellular-signal-regulated kinase (ERK), but not the activation of protein kinase B (Akt) [67]. According to the literature of melanoma biology, melanoma cellular functions and stemness characteristics are critically controlled by Ras-Raf-MEK-ERK and PI3K-Akt pathways [73]. Highly activated Src-signaling also critically involves in melanoma progression [74]. Highly activated Src phosphorylates β-catenin on tyrosine 654 (Y654) thereby triggering β-catenin release from adherens junctions and increasing the level of β-catenin in plasma membrane [75]. Similarly, over-activated ERK inhibits the expression of E-Cadherin [76]. Therefore, the silenced Prx II expression in melanoma disrupts the E-Cadherin-β-catenin complex via overactivation of Src and ERK signaling, thereby increasing epithelial-mesenchymal transition (EMT) and stemness characteristics of melanoma including cell proliferation, migration and lung metastasis (Figure 5) [67].

Thus, Prx II has been suggested as a therapeutic target for melanoma to inhibit melanoma metastasis, because of the ability of Prx II to induce stabilization of adherens junctions and thereby to reduce EMT and metastasis [67].

## 6. Prx II Protects Radioresistant and Metastatic Breast Cancer Stem Cells from Oxidative and Metabolic Stress via Metabolic Adaptation

Breast cancer is a most common malignancy and it is considered to be the leading cause of cancer-related death in women worldwide [77]. Radiotherapy is one of the commonly applied therapies after breast-conserving surgery as a standard combination therapy for breast cancers [78]. But the side effects associated with radiotherapy and acquired radioresistance of breast cancers make radiotherapy is non-beneficial for some patients who received radiotherapy treatments [79]. Accumulating evidence suggested that CSCs with stemness properties are responsible for radioresistance in breast cancers. Furthermore, studies reported that residual breast CSCs after radiotherapy obtain radioresistance thereby increasing stem cell population after treatments [80,81].

**Figure 6:**
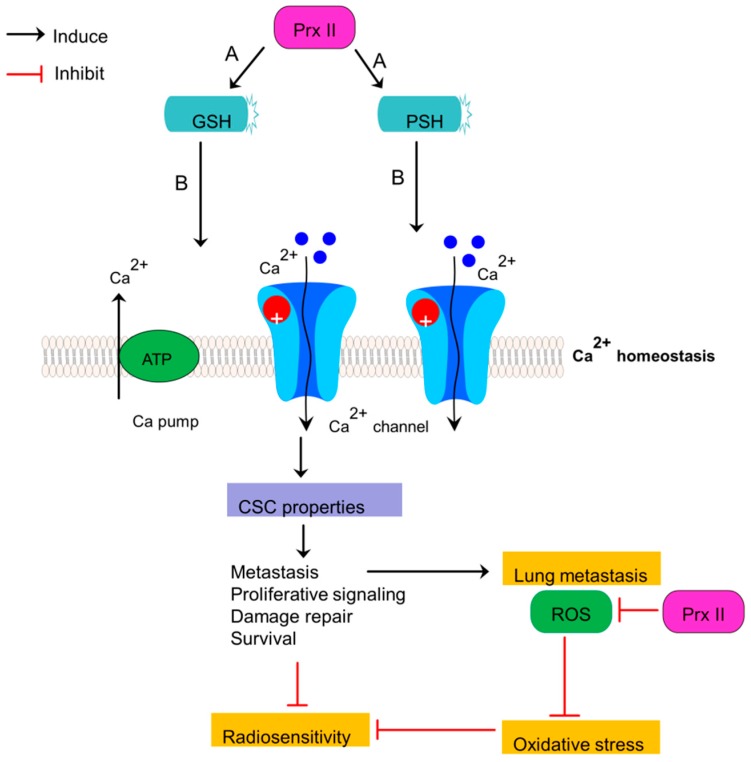
Stemness-associated properties and radiosensitivity are mediated by Prx II in breast cancers. Up-expression of Prx II in breast cancers induces the consequence expression of protein thiol (PSH) and glutathione (GSH). These expressions are associated with decreased cellular toxicity by induced Ca^2+^ efflux and Ca^2+^ homeostasis. Cellular toxicity reduction induces the stemness-associated properties of CSCs and decreases the sensitivity to radiotherapies. Prx II also protects lung metastatic breast cancer cells from ROS-mediated oxidative stress in lung microenvironment.

Radioresistant breast CSCs show acquired abilities to survive against anti-cancer treatments over sensitive cancer cells. One of the mechanisms by which those stem cells obtain radioresistance is activation of various antioxidative systems [35]. Prx II is also a member of aforementioned antioxidant network [82]. High Prx II expression in breast CSCs compared with non-cancerous cells is believed to be associate with the development of radioresistant breast cancer cells, greater cell proliferation, anti-apoptotic properties, differentiation, intracellular signaling and gene expression [18,83,84]. Generation of high level of ROS by Ionizing Radiation, personalized radiotherapy and chemoresistant agents is one of major mechanisms to obliterate cancer cells by damaging DNA, activating transcription factors, their downstream pathways and cytotoxic programs [85,86]. But, breast CSCs with overexpressed Prx II sustain cells against radical damages by deleterious effects of ROS and result radioresistance and chemoresistance in breast cancers. Prx II induces the survival rate of breast cancer cells by restoring redox balance [35]. The mechanism by which Prx II catalyze ROS scavenging and detoxify cells against oxidative stress is an enzyme-substrate reaction which is acquired by reducing peroxides via cysteine residue in the active site. Active sites of Cys residues (Peroxidative Cys, Resolving Cys) in Prx II oxidize to a dimer by forming internal and external disulfide linkage and sulfenic acid is followed by the reduction of peroxides as the substrate of Prx II enzyme [35]. Therefore, the expression of Prx II correlates with resistance to radiation-induced apoptosis by eliminating detrimental effects of ROS [25]. Breast CSC membrane is believed to be protected by Prx II against radiation damages as Prx II is associated with cell membrane as a response to oxidative stress, even though it is a cytosolic protein [83,87]. Prx II induces glutathione (GSH) and protein thiol (PSH) levels and thereby decreasing radio sensitivity of breast CSCs via reducing cellular toxicity and protecting Ca^2+^ homeostasis, because the membrane association of Prx II links with Ca^2+^-activated K^+^ channel (Figure 6) [35,88]. Therefore, sensitivity of breast cancers to radiotherapy is increased by forced silencing of Prx II gene expression as reported in a previous study [81].

Prx II is also reported to express highly in metastatic breast cancer cells in lungs [89]. Lung is the second target of metastatic breast cancer cells, whereas bones rank first [90]. Usually, most of metastatic cells are destroyed in lungs by a mechanism which activates alveolar macrophages [91], because metastatic cells have to suffer from different oxygen pressure in lungs than other systems [92]. But, highly expressed Prx II in metastatic breast cancer cells effectively scavenge intracellular ROS thereby stabilizing redox state suitable for cell survival, colonization and protecting cells against lung microenvironment [89]. Therefore, studies have suggested that combination of radiotherapy with alteration of Prx II expression levels may be a promising approach to selective killing of radioresistant breast CSCs and lung metastatic breast cancer cells [18,35,89].

## 7. Prx II Maintains CSC Phenotype in Colorectal Cancer via Regulating Wnt/β-Catenin and Hedgehog Signaling Pathways

Colorectal cancer is the third leading cause of cancer related mortality worldwide [93]. Prx II is expressed highly in colon CSCs and normal colon cancer cells compared with normal colon cells [12,19,31,94,95]. Prx II induces the CSC-related properties such as expression of stemness genes, cell growth, cell survival, differentiation, stem cell self-renewal and metastasis in advanced colon CSCs via activating Hedgehog (HH)/Gli1 signaling pathway [31]. Prx II is also involved in the Wnt/β-Catenin signaling in colorectal cancers. Wnt signaling requires for normal stem cell maintenance, whereas tumor development is linked with dysregulated Wnt signaling [19]. Prx II also co-relates with the stage of cancer, lymph node metastasis, cell proliferation, drug resistance and colon cancer progression and negatively relates with the disease free survival (DFS) and disease specific survival (DSS) of colon cancers via Wnt signaling [19]. A previous study has shown that Prx II over activates Wnt signaling which then inhibits the activities of glycogen synthase kinase 3 beta (GSK-3β) through activated Dishevelled (Dsh) protein. Inactivation of GSK-3β induces the translocation of β-Catenin to nucleus and transcription of lymphoid enhance factor/T-cell factor (LEF/TCF) target genes such as survivin and c-Myc. Overexpression of those genes enhances the cell survival and stem cell related properties and inhibit cell apoptosis. Furthermore, inactivation of GSK-3β inhibits the phosphorylation of β-Catenin and thereby reducing degradation signaling [19].

**Figure 7:**
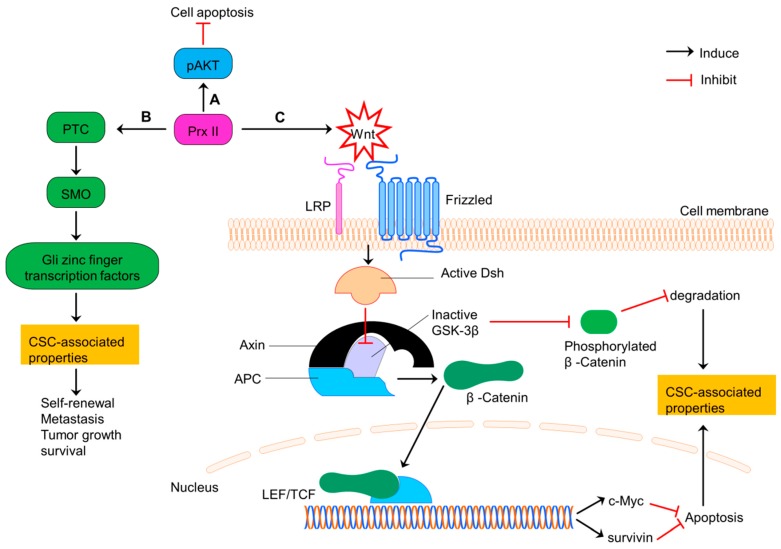
Prx II regulates CSC properties of colorectal cancers via PI3K/AKT, Hedgehog and Wnt/β-Catenin signaling. (**A**) Induced PI3K/AKT signaling by overexpressed Prx II in colorectal cancer cells to increase cell survival by inhibiting apoptosis. (**B**) Prx II-mediated overactivation of Hedgehog pathway by increasing the expressions of PTC, SMO and Gli zinc finger transcription factors to promote stemness-associated properties. (**C**) Activation of Wnt pathway by overexpressed Prx II. Wnt signaling induces translocation of β-Catenin to nucleus by activated Dishevelled and inactivated GSK-3β activities. Translocated β-Catenin induces the transcription of LEF/TCF complex target genes such as survivin and c-Myc. Activation of these genes and deactivation of degradation signals maintains the stemness-associated properties of colorectal cancers.

Inactivation mutations of the adenomatous polyposis coli (APC) tumor suppressor gene are common among more than 50% of colon cancer patients. Those APC mutations induce the initiation of intestinal tumorigenesis via inducing transcriptionally active β-Catenin accumulation, because APC is considered as the major scaffold protein in β-Catenin destruction complex [96,97]. APC mutation causes post transcriptional modifications of Prx II and those modifications result conformational changes in Prx II which associate with the protein-protein interaction of Prx II with tankyrase (TNKS), an enzyme belongs to the poly (ADP-ribose) polymerase (PARP) family enabled by Gly residue protrusion in Prx II structure [98]. Through this direct binding via ARC4/5 domains in the cytosol, Prx II protects TNKS against the oxidative inactivation and preserves the deregulated β-Catenin pathway in APC-mutant colon cancer cells with high endogenous H_2_O_2_ expression. It has been reported that the absence of Prx II increases the disruption of transcriptionally active β-Catenin in colon cancer cells through canonical destruction complex (Figure 7). Thus, Prx II is necessary for APC-mutation govern tumorigenesis in intestine [98,99].

Previous study has also showed that, forced knockdown of Prx II suppresses PI3K/AKT signaling pathway and increases the sensitivity of colon cancers to 5-FU. Thus, targeting Prx II have potential therapeutic effects to treat colorectal cancers with and without APC mutations as it is already proved the increasing sensitivities of colon cancers to existing chemo and radiotherapy by inhibiting Prx II expression [94,100].

## 8. Loss of Prx II during Bladder Cancer Progression is Associated to the Induction of Stemness Characteristics

Bladder cancer is considered as one of the most common malignancies of the urinary system [101]. A previous study has reported that Prx II is expressed highly in bladder cancer urine specimens compared with non-cancerous specimens [102]. But, Prx II expression in bladder cancers seems to vary based on the stage and differentiated grade of cancer. The expression of Prx II is significantly reduced in more malignant, advanced bladder cancer cells compared with early stage non-invasive bladder cancer cells, indicating the loss of Prx II during bladder cancer progression and tumor development [39,42,103]. This has been reported in a previous study by showing the higher Prx II expression in noninvasive papillary carcinoma than the tumors that spread to connective tissues, muscle of the bladder wall, peri vesical tissue, abdominal wall, pelvic wall, prostate or seminal vesicle and uterus or vagina [39]. Therefore, the reduction of Prx II considered to be involved with cancer stemness characteristics including metastasis and tumor progression [42].

But, it is yet to be studied more in details about the expression levels of Prx II in urinary bladder carcinoma and the association of those Prx II with CSC phenotype and stemness-associated properties depending on the differentiated grade.

## 9. Prx II Reduces Radiosensitivity and Oxidative Stress of Glioma

Glioma is the most commonly diagnosed heterogenous group of tumor in central nervous system which is responsible for the majority of malignancies [104]. The level of Prx II is reported to be increased in glioma cells compared with normal astrocytes [105]. Previous study has reported that Prx II significantly induces the survival rate of glioma and positively associates with grade of glioma malignancy [106]. Overexpression of Prx II protects glioma cells against ROS-induced oxidative stress and thereby against existing chemo and radiotherapies. Similarly, a study has shown that silencing of Prx II sensitizes glioma cells to anticancer therapies [105].

**Figure 8:**
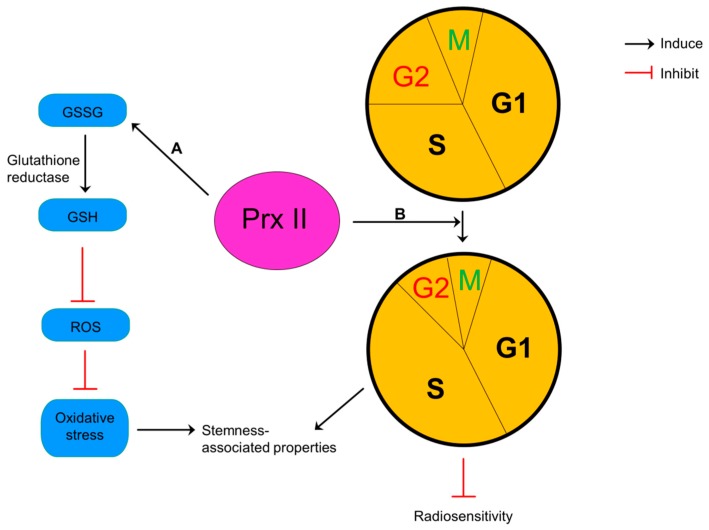
Pivotal role of Prx II in glioma cells. (**A**) Overexpression of Prx II in glioma cells increases the reduction of oxidized glutathione (GSSG) to reduced glutathione (GSH) by induced glutathione reductase activity thereby reducing ROS level and damages from oxidative stress and inducing stemness-associated properties. (**B**) Prx II-mediated alteration of cell cycle distribution by decreasing cells in most radiosensitive G2/M phase whereas increasing cells in most proliferative S1 phase. Alterations of cell cycle result poor radiosensitivity and induced stemness-associated properties.

Reduction of intracellular glutathione (GSH) by inhibited activities of glutathione reductase and alterations in cell cycle distribution have been presented as two possible mechanisms to explain this sensitization of glioma cells to anticancer therapies by silencing of Prx II [105]. Glutathione usually exists both in oxidized (glutathione disulfide-GSSG) and reduced (GSH) states and the ratio of GSH:GSSG considers as an indicator for cellular redox environment [107]. Silencing of Prx II results oxidized environment by reducing the ratio between reduced and oxidized glutathione in glioma cells and inhibiting the reduction of GSSG to GSH by decreased activities of antioxidant enzyme, glutathione reductase. Glioma cells increase the sensitivity to the oxidative stress in this oxidized environment [105]. Reduction of Prx II level in glioma cells also alters the cell cycle distribution by decreasing cells in more proliferative phase (S) and increasing cells in most radiosensitive phase (G2/M) and increases the cell cycle doubling time (Figure 8). These evidences show that Prx II induce cell proliferation and resistance to oxidative resistance and radiotherapy glioma cells. Therefore, silencing of Prx II sensitizes glioma cells to oxidative stress and radiotherapy by inhibiting cell proliferation [105].

## 10. Prx II Promotes Prostate Cancer Progression via Distinct Regulation of AR Transactivation

**Figure 9:**
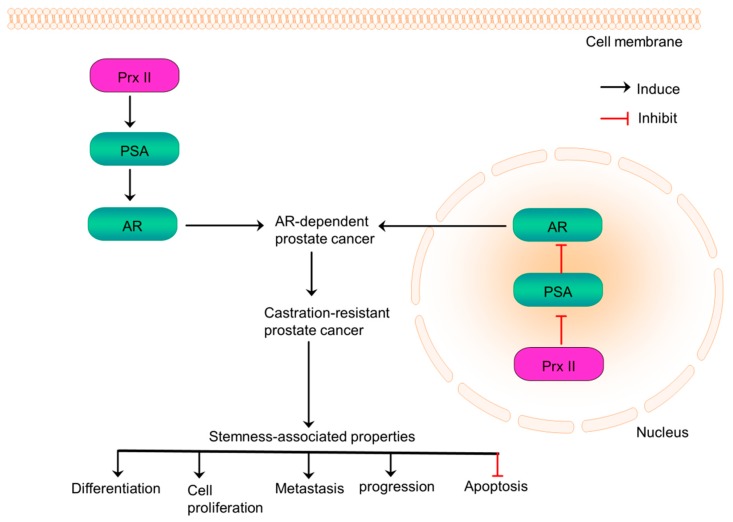
Prx II-mediated prostate cancer progression promotes stemness-associated properties. Prx II is localized both in cytosol and nucleus in AR-expressing prostate cancer cells and castration-resistant prostate cancer (CRPC) cells. Overexpression of Prx II in cytosol induces AR transactivation whereas inhibits in nucleus. Distinct regulation of AR transactivation induces the growth of AR-expressing prostate cancers, stemness-associated properties and formation to CRPCs.

Prostate cancer is the most commonly diagnosed cancer among men and is responsible for the most cancer-related deaths in men in the world [20,108]. Prostate cancer progression is known to be regulated by androgen/androgen receptor (AR) signaling pathway [20]. AR signaling associates with multiple cellular functions related to stemness such as proliferation, metastasis, differentiation and apoptosis [109]. Overexpressed AR signaling develops resistance of prostate cancers to existing anti-androgen treatments [110]. Prx II is considered as a key factor which regulates AR signaling in prostate cancers. Prx II is overexpressed and localized to both cytoplasm and nucleus in AR-expressing prostate cancer cells. Those overexpressed Prx II in nucleus suppresses the transactivation of AR by reducing prostate specific antigen (PSA) transcription and Prx II in cytoplasm increases AR transactivation by inducing PSA transcription (Figure 9). It has been reported that Prx II expression-affected another gene or Prx II-altered oxidative stress in intracellular fractions may control the transactivation of Prx II [20].

Prx II-mediated distinct regulation of AR transactivation increases AR-expressing prostate cancer cell proliferation, cell growth and the progression of AR-expressing prostate cancers to castration-resistant prostate cancer (CRPC). A study showed that Prx II knockdown affects to prostate cancer cell growth via AR signaling by presenting the evidence that Prx II inhibits growth only in AR-expressing prostate cancer cells but not in AR-null cells [20]. Interestingly, the expression of other Prx family members is increased by Prx II knockdown without any influence on the cell growth. Prx II knockdown also suppresses the expression of androgen-regulated genes such as PSA. Thus, Prx II is a therapeutic target for AR-expressing prostate cancers to inhibit its progression to CRPC and to suppress stemness-related characteristics [20]. But, Prx II upregulation and localization mechanisms and the mechanisms by which Prx II affects AR-target genes and Prx isoforms in AR-expressing prostate cancers are yet to be further explored.

## 11. Prx II Suppresses Leukemogenesis and the Stem Cell Maintenance of Acute Myeloid Leukemia Blasts

Acute myeloid leukemia (AML) is an aggressive cancer begin in myeloid line of blood cells and cause fast growth of abnormal blood cells. Prx II expression is lower in AML blasts than normal progenitor cells and white blood cells [5]. Prx II was identified as an epigenetically silenced tumor suppressor gene in AML in a previous study [5]. Mutual dependence between decreased level of histone H3 acetylation and DNA hypermethylation at Prx II gene promoter causes the silenced expression of Prx II mRNA and protein in AML. Often, epigenetically silenced tumor suppressors characterized by a low level of histone acetylation and higher DNA methylation [5,111]. Silenced levels of Prx II in AML blasts induce ROS level within stem cells rather than its microenvironment and maintain stem cell state which then contributes to develop leukemia [5]. Also, silencing of Prx II in AML blasts stimulates interleukin 3 (IL-3) cytokines, thereby increasing the phosphorylation of MAP kinase to induce downstream signaling pathways including ERK signaling. Activated signaling pathways play a role in maintenance of stemness-associated characteristics [5].

**Figure 10:**
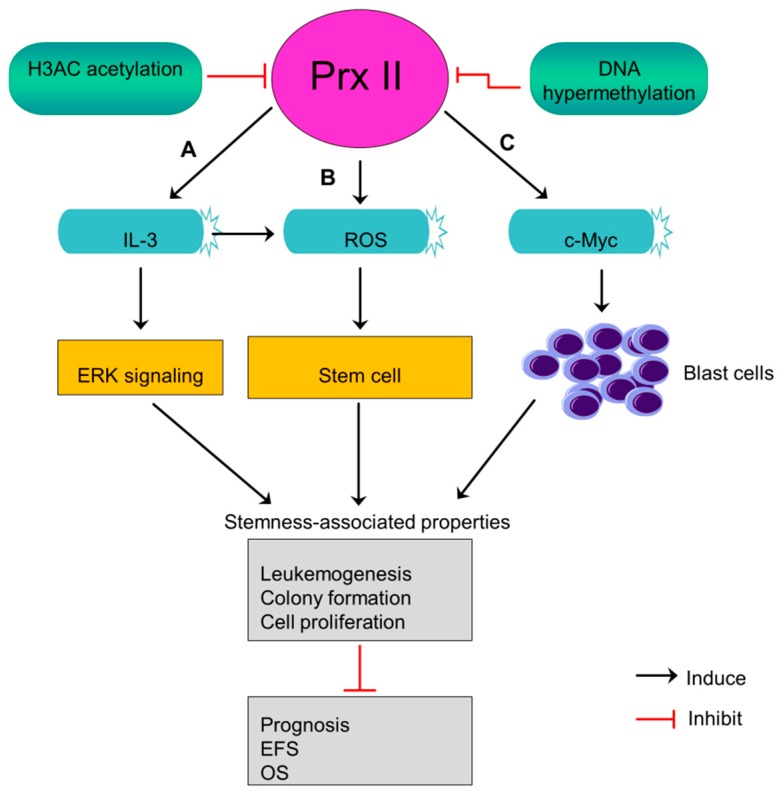
Epigenetically silenced Prx II induces stem cell maintenance in AML. (**A**,**B**) Epigenetically silenced Prx II in AML by DNA hypermethylation and histone (H3AC) acetylation increases ROS level and stimulates interleukin 3 (IL-3) to induce stemness-associated properties. (**C**) Silencing of Prx-induced c-Myc-dependent leukemogenesis by increasing blast cells. Consequent promotion of stemness-related properties thereby reducing prognosis, event free survival (EFS) and overall survival (OS) of AML patients.

AML patients with lacked Prx II have been reported to shorten their event-free survival (EFS) rate and overall survival rate (OS), indicating that decreased Prx II protein expression in AML blasts causes poor prognosis. Moreover, epigenetically silenced Prx II in AML blasts promotes c-Myc induced-leukemogenesis by increasing the number of blast cells (Figure 10) [5]. A study has proved Prx II as inhibitor of myeloid cell growth by showing the effects of forcedly overexpressed Prx II on myeloid cell growth. Thus, forced expression of Prx II in blast cells may have potential effects to overcome AML. But, the mechanism by which Prx II exerts its tumor suppressor functions is not fully studied so far.

## 12. Association of Prx II with Stemness Properties in Cancer below Should Be Studied in the Future

### 12.1. Osteosarcoma

Osteosarcoma is the most common primary malignant bone cancer among adolescents and children worldwide [112]. Expression of Prx II in osteosarcoma is inversely related with the response to chemotherapy, as Prx II is highly expressed in poor responders compared with good responders [113]. Furthermore, a study reported that silencing of Prx II sensitizes osteosarcoma cells to existing chemotherapeutic drugs and inhibits the stemness-related properties such as cell proliferation, invasion, migration and metastasis [40]. Thus, Prx II is considered to be a responsiveness biomarker for osteosarcoma patients because of the potential value of Prx II to predict response to chemotherapy [40].

### 12.2. Head and Neck Cancers

Head and neck cancers consist of a wide range of cancers including oral cavity and oropharyngeal cancers, nasopharyngeal cancers and laryngeal and hypopharyngeal cancers [114]. Prx II has been reported to express highly in those cancers and protect head and neck cancer cells from oxidative stress and radiation-induced cellular damages. Level of Prx II also has been reported to increase further in head and neck cancer cells after radiotherapy. Therefore, studies suggest that the inhibition of Prx II and introduction of Prx II antisense may sensitize head and neck cancer cells to existing chemo and radiotherapies [25].

### 12.3. Renal Cell Carcinoma

High level of Prx II is believed to be associate with the formation of aggressive tumors in renal cell carcinoma as abundantly expressed Prx II in low grade tumors and tumors with fewer metastases protects the renal cell carcinoma cells from oxidative damages activates the pathways [115].

### 12.4. Lymphoma

Lymphoma is heterogeneous blood cancer group develop from lymphocytes which further divides mainly into Hodgkin’s lymphoma and non-Hodgkin lymphoma. It has been reported that Prx II is silenced in classical Hodgkin lymphoma (cHL) due to the hypermethylation and transcriptional silencing of Prx II [116]. But, Prx II is considered to be upregulated in human Burkitt lymphoma which is categorizing under non-Hodgkin lymphoma. A study showed that forcedly silencing of Prx II significantly reduces cell proliferation, survival and growth rate in Burkitt lymphoma cells, and indicating Prx II-induced stemness properties of non-Hodgkin lymphoma [117].

### 12.5. Ovarian Cancer

Ovarian cancer is the most common cause of gynecological cancer-associated deaths in women worldwide [37]. Prx II is expressed higher in malignant borderline ovarian tumors than non-cancerous benign tumors [118]. Prx II is also reported to induce during ovarian carcinogenesis there by protecting ovarian cancer cells from oxidative stress and detrimental effects [36,119].

### 12.6. Pancreatic Cancer

Pancreatic cancer is one of the deadliest solid malignancies which acquire resistance against existing therapeutic drugs including gemcitabine [120]. Prx II is significantly increased in gemcitabine-resistant pancreatic cancer cells compared with gemcitabine-sensitive pancreatic cancer cells. Thus, Prx II is a potential biomarker to predict the sensitivity of pancreatic cancer patients to gemcitabine [38].

## 13. Conclusions

Peroxiredoxin II (Prx II) is an antioxidant enzyme with cell-type dependent expression in cancers [32]. Overexpressed or silenced Prx II is reported to maintain CSC phenotype, redox homeostasis and stemness-associated properties of cancer cells. Careful consideration of the literature on Prx II-based studies on various cancers revealed that Prx II is overexpressed in hepatocellular carcinoma, non-small cell lung cancer, breast cancer, colorectal cancer, glioma and prostate cancer. Overexpression of Prx II is associated with CSC phenotype and stemness-associated properties of aforementioned cancers [20,21,22,39,86,91]. Even though osteosarcoma, head and neck cancer, renal cell carcinoma, ovarian cancer, pancreatic cancer, cervical cancer secretome and malignant mesothelioma also were identified as cancers with higher Prx II expression, further studies are required to observe its association with CSCs and stemness-associated properties [121,122]. Gastric cancer, melanoma and leukemia are identified as cancers with silenced Prx II by aberrant CPG promoter methylation and histone acetylation in Prx II gene. Silenced Prx II is correlates with CSC phenotype and stemness-associated characteristics of those cancers [5,24,41]. Furthermore, bladder cancer shows relatively higher Prx II expression in early stages and it reduces significantly with bladder cancer progression to malignant stages. Expression of Prx II in lymphoma further depends on the lymphoma cell type because, Prx II is silenced in Hodgkin lymphoma but not in Burkitt lymphoma which belongs to non-Hodgkin lymphoma [114,115].

Prx II regulates stemness-associated characteristics of cancers via several pathways, mainly including VEGF/VEGFR/STAT3, Ras/FoxM1, JNK, Wnt/β-Catenin, Hedgehog and androgen (AR)/androgen receptor signaling [19,20,21,22,43,91]. Prx II also contributes to the resistance of cancer cells to existing chemo and radiotherapies by regulating redox statues and maintaining redox homeostasis. Therefore, we believe that forceful alteration of Prx II expression may increase therapeutic efficacy of current cancer-therapies through minimized stemness and related characteristics and maximized oxidative stress. It may provide a potential to eradicate CSCs with higher stemness, which believed to be remained after treatments and cause recurrence of cancers.
